# Simultaneous Detection of Dopamine and Uric Acid Using a Poly(l-lysine)/Graphene Oxide Modified Electrode

**DOI:** 10.3390/nano6100178

**Published:** 2016-09-28

**Authors:** Yuehua Zhang, Wu Lei, Yujuan Xu, Xifeng Xia, Qingli Hao

**Affiliations:** 1 School of Chemical Engineering, Nanjing University of Science and Technology, Nanjing 210094, China; zhangyuehua969@126.com (Y.Z.); nlgpomm@163.com (Y.X.); lethe120@163.com (X.X.); 2 School of Chemistry and Chemical Engineering, Nantong University, Nantong 226007, China

**Keywords:** graphene oxide, poly(l-lysine), dopamine, uric acid, simultaneous determination

## Abstract

A novel, simple and selective electrochemical method was investigated for the simultaneous detection of dopamine (DA) and uric acid (UA) on a poly(l-lysine)/graphene oxide (GO) modified glassy carbon electrode (PLL/GO/GCE) by differential pulse voltammetry (DPV). The electrochemically prepared PLL/GO sensory platform toward the oxidation of UA and DA exhibited several advantages, including high effective surface area, more active sites and enhanced electrochemical activity. Compared to the PLL-modified GCE (PLL/GCE), GO-modified GCE and bare GCE, the PLL/GO/GCE exhibited an increase in the anodic potential difference and a remarkable enhancement in the current responses for both UA and DA. For the simultaneous detection of DA and UA, the detection limits of 0.021 and 0.074 μM were obtained, while 0.031 and 0.018 μM were obtained as the detection limits for the selective detection of UA and DA, using DPV in the linear concentration ranges of 0.5 to 20.0 and 0.5 to 35 μM, respectively. In addition, the PLL/GO/GCE demonstrated good reproducibility, long-term stability, excellent selectivity and negligible interference of ascorbic acid (AA). The proposed modified electrode was successfully implemented in the simultaneous detection of DA and UA in human blood serum, urine and dopamine hydrochloride injection with satisfactory results.

## 1. Introduction

Dopamine (3,4-dihydroxyphenylethylamine, DA) is one of the most important neuro-transmitters that plays an important role in human metabolism [1]. The deficiency of DA content may be related to the emergence of clinical illnesses, such as Parkinson’s disease, Schizophrenia [2]. DA can be supplied as a medicine, and its overdosage may increase the heart rate and blood press. Considering the importance of DA in physiology and pathology, it is necessary to develop highly efficient and sensitive methods to detect DA. In recent years, many approaches have been investigated to detect DA, including spectrophotometric [3], chemiluminescent [4], fluorescent [5], liquid chromatographic [6], and electrochemical methods [7,8]. The physiological level of DA is very low, and is in the range of 0.01–1.0 μΜ [9]. Some other important neuro-transmitters, such as epinephrine, norepinephrine, serotonin and so on, always coexist in the central nervous system. Therefore, in vivo and vitro detection of dopamine with high sensitivity and selectivity is of great significance. Fast scan cyclic voltammetric with carbon-fiber implantable biosensors have been reported for in vivo analysis of DA [10].

Uric acid (2,6,8-trihydroxypurine, UA) is the primary end product of purine metabolism. Average level of UA in blood is about 120–450 μM and in urine is about 2 mM. Abnormal levels of UA in human body fluids may lead to the occurrence of gout, hyperuricemia or Lesch-Nyhan syndrome [11]. Thus, the detection of UA has the vital significance in the field of clinical diagnosis. Several methods, such as enzymatic [12], voltammetric [13], high performance liquid chromatographic [14], and phospho-tungstic acid deoxidizing methods, have been utilized for the detection of UA. Because DA and UA always coexist in human biological fluids, it is highly desirable to propose a selective, sensitive and simultaneous detection technique for analytical research and diagnostic applications Electrochemical methods with advantage of sensitivity, rapidity and low cost have been widely adopted for the simultaneous detection of UA and DA [15,16,17]. However, three major problems will be encountered at the bare solid electrode. Firstly, the concentration of DA is much less than that of UA in biological samples. Secondly, the sensitivities to DA and UA are low. Thirdly, DA and UA often coexist with ascorbic acid (AA) in real biological samples and all of them are electrically active substances. Moreover, DA and AA exhibit nearly identical redox peaks [18]. Therefore, the presence of AA will interfere with the simultaneous determination of DA and UA. Therefore, it is difficult to realize the simultaneous detection of DA and UA on the bare electrode.

In order to tackle this issue, many kinds of materials are used to modify the electrode to raise the sensitivity and selectivity in the simultaneous detection of DA and UA [19,20,21]. Among these methods, conducting polymer-modified electrodes, one of the most important electrodes, have been fabricated as electrochemical sensory platforms for the detection of analytes because of their high selectivity, sensitivity, homogeneity, and chemical stability along with strong adherence of the polymer film [22]. For example, poly(acrylic acid)-multi-walled carbon-nanotube composites [23], poly(vinyl alcohol) [24], poly(sulfonzto III) [25]-modified electrodes, etc., were studied in the simultaneous detection of DA and UA. Poly(l-lysine) (PLL), because of its good biocompatibility and flexible structure framework, is a well-known biologically friendly polymer that has gained increasing interest, especially when combined with graphene or graphene oxide [26]. The combination of both materials can offer a biocompatible environment for the following functionalization or the adsorption of biomolecules. Therefore, some biosensors have been fabricated based on PLL-graphene composites for glucose [27], H_2_O_2_ [28,29], cancer cells [30] and genes [31]. Although studies on the detection of DA or AA based on PLL-modified electrodes have been previously published [32,33], no work has been reported on the application of PLL/GO-modified electrodes for simultaneous electrochemical studies of DA and UA.

Herein, we report an electrochemical analytical method for the fast, simultaneous detection of DA and UA based on a PLL/GO-modified glassy carbon electrode (PLL/GO/GCE). The sensory platform of the PLL/GO/GCE was constructed by electropolymerization of l-lysine onto the GO-modified GCE in the potential range of −0.8~2.0 V through cyclic voltammetry (CV). Compared to the electrochemical performances of UA and DA at various electrodes, including the PLL-modified GCE (PLL/GCE), untreated GO-modified GCE (untreated-GO/GCE), the modified electrode with GO scanned from −0.8 to 2.0 V for 10 cycles at 50 mV·s^−1^ (GO/GCE), and the bare GCE, the excellent electrochemical properties at the PLL/GO/GCE were observed as a result of the superior electrochemical activity of the PLL/GO film toward UA and DA. The high electrocatalytic activity of PLL/GO/GCE to the redox reaction of UA and DA is attributed to the good combination of PLL and GO and the synergistic effect of both composites. Fabrication of a novel electrochemical sensor based on the PLL/GO composite film can provide a facile and effective approach to the simultaneous detection of UA and DA.

## 2. Results and Discussion

### 2.1. Fabrication and Characterization of Poly(l-lysine)/Graphene Oxide Modified Glassy Carbon Electrode (PLL/GO/GCE)

**Figure 1:**
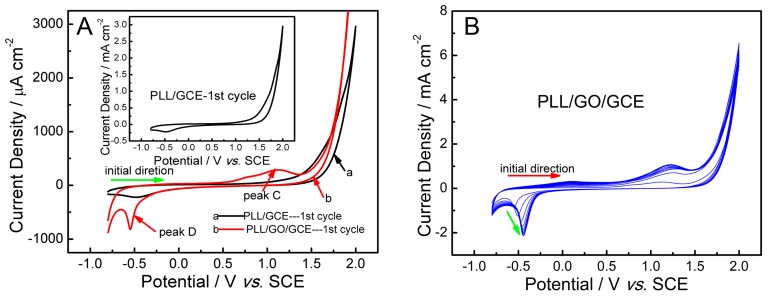
(**A**) The first cycle of CVs- for the polymerization of 10 mM l-lysine in 0.1 M phosphate buffer solution (PBS, pH 8.0) at glassy carbon electrode (GCE) (a) and untreated- graphene oxide (GO)/GCE (b). Scan rate: 50 mV·s^−1^. Inset is curve (a); (**B**) CVs of untreated-GO/GCE recorded during electropolymerization of 10 mM l-lysine in 0.1 M PBS (pH 8.0). Scan rate: 50 mV·s^−1^. The first 10 consecutive scans were depicted.

The first cycles of the cyclic voltammograms (CVs) are recorded for the electropolymerization of 10 mM l-lysine in 0.1 M phosphate buffer solution (PBS, pH 8.0) at the bare GCE and the untreated-GO/GCE, respectively (Figure 1A). As can be seen in Figure 1A, for the bare GCE, no visible anodic peaks are found in the first cycle. The oxidation onset potential of l-lysine appears at approximately +1.0 V and a weak cathodic peak appears at −0.46 V in the reverse cathodic scan. For the untreated-GO/GCE, a pair of apparent redox peaks is observed at the anodic peak potential of +1.125 V (peak C) and the cathodic peak potential at approximately −0.51 V (peak D), indicating the oxidation and reduction of l-lysine, respectively. The oxidation onset potential (+0.6 V) of l-lysine at the untreated-GO/GCE is much lower than that at the GCE, indicating that the GO sheets as the connecting matrix for PLL can remarkably reduce the oxidation potential of l-lysine. The remarkable redox peaks and lower oxidation onset potential of l-lysine indicate the increased electrochemical polymerization activity of l-lysine at the untreated-GO/GCE. Furthermore, as shown in Figure 1A, the background current at the untreated-GO/GCE is higher than that at the GCE, which is attributed to the high specific surface area of GO. The results suggest that the untreated-GO sublayer is in favor of the nucleation and growth of PLL.

Figure 1B depicts the first 10 consecutive scans of the polymerization at the untreated-GO/GCE. The redox peak currents increased with successive scanning, which reflects the continuous growth of the film. However, the peak currents increased slowly up to the 10th cycle, implying that the surface of the untreated-GO/GCE was generally covered with PLL film over ten cycles. Meanwhile, the sublayer of GO was also overoxidized.

**Figure 2:**
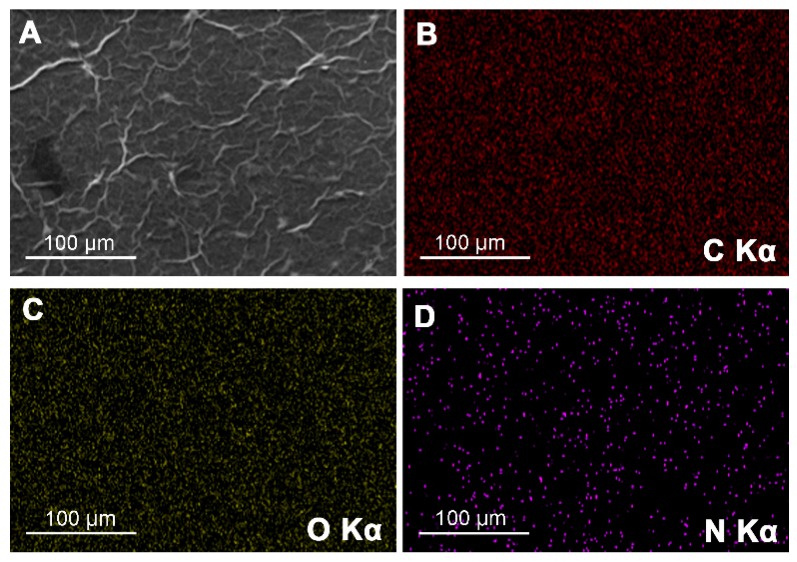
SEM images of the poly(l-lysine)/GO composite on the GCE (**A**) and the corresponding X-ray spectroscopy (EDS) element mapping of C (**B**); O (**C**) and N (**D**).

Figure 2A shows the SEM image of PLL/GO composite, which indicates that the PLL is uniformly deposited onto the surface of the untreated-GO/GCE, and the surface of PLL/GO/GCE presents the typical features of GO with wrinkled paper-like sheets. This result is further supported by energy dispersive X-ray spectroscopy (EDS) element mapping (Figure 2B–D). The EDS spectra of PLL/GO clearly illustrate the presence of carbon, oxygen and nitrogen in the PLL/GO composite, which also confirms the electropolymerization of l-lysine on the GO-modified GCE.

### 2.2. Electrochemical Behaviors of Uric Acid and Dopamine at Various Electrodes

**Figure 3:**
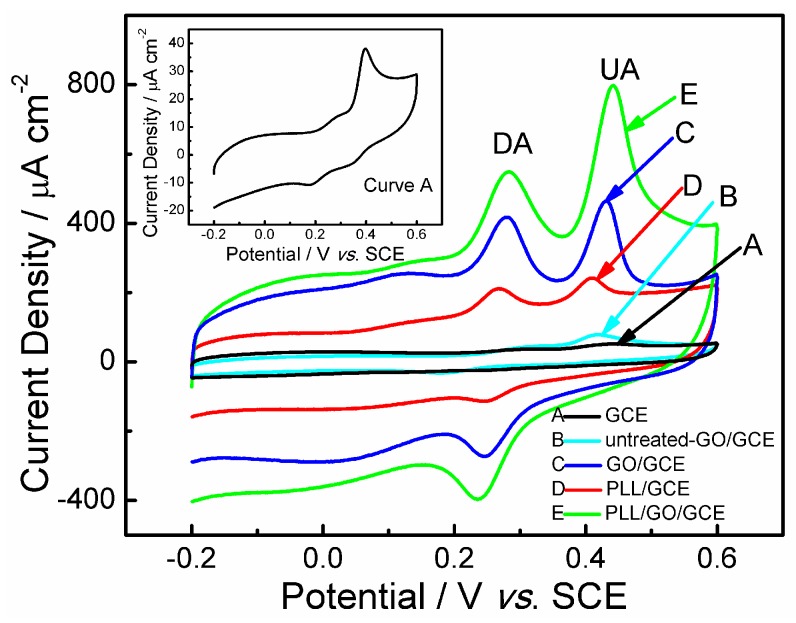
CVs of 1.0 × 10^−4^ M UA and 1.0 × 10^−5^ M dopamine (DA) in citric acid-phosphate buffer solution (CPS, pH 5.0) at the bare GCE (**A**); untreated-GO/GCE (**B**); GO/GCE (**C**); poly(l-lysine) PLL/GCE (**D**) and PLL/GO/GCE (**E**). Scan rate: 100 mV·s^−1^. Potential window: –0.2~0.6 V. Inset: Curve A.

The electrochemical behaviors of DA and UA at various electrodes were investigated by CV in citric acid-phosphate buffer solution (CPS, pH 5.0). The results are shown in Figure 3. Compared to the CVs at the modified electrodes, the electrochemical behaviors of UA and DA at the bare GCE are very weak with an overlapped anodic peak. UA shows a weak oxidation peak at 398 mV and no reduction peaks, whereas a pair of very weak redox peaks of DA is presented (Curve A, inset of Figure 3). The weak electrochemical property and irreversible redox reactions of DA and UA suggest the sluggish electron transfer kinetics of DA and UA at the bare GCE, which may result from the deposition of the oxidation products of DA and UA on the electrode surface [19]. Thus, it is hard to separate DA and UA using the CV technique at a bare GCE.

The GCE modified with untreated-GO (Curve B) shows the improved peak current densities towards UA and DA attributed to the higher specific surface area of GO, but only a pair of weak redox peaks of DA and one oxidation peak of UA were observed. Therefore, the reactions of both UA and DA are still irreversible at the untreated-GO/GCE. Compared with the bare and untreated-GO modified GCEs, all peak currents at the PLL/GCE increased (Curve D). However, the GO/GCE (Curve C), where the GO was scanned from −0.8 V to 2.0 V for 10 cycles at 50 mV·s^−1^, shows one pair of redox peaks of DA and one oxidation peak of UA. Moreover, the peak currents of analytes at the GO/GCE are much larger than those at untreated-GO/GCE (Curve B) and PLL/GCE (Curve D), but still smaller than those at the PLL/GO/GCE (Curve E). It indicates that the treated GO shows better electrocatalysis to the redox reactions of DA and UA than the untreated-GO and PLL. It may be due to the over-oxidation of GO at the high potential, leading to the more oxygen-containing groups produced in carbon sheets. The more oxygen-containing groups are beneficial for the adsorption of UA and DA through hydrogen bonds. In our previous work [34], the similar activation of carbon materials at high potential could improve the electrocatalysis of electrodes imidacloprid.

For the PLL/GCE (Curve D), the peak current density of UA exhibits approximately 2.5 times enhancement compared to that at the untreated-GO/GCE. The enhanced peak current density and the improved electrochemical activities toward DA and UA can be attributed to the good conductivity and the more active adsorption sites of PLL, which are prone to combination with nitrogen or oxygen atoms of analytes through hydrogen bonding interactions [35]. With the combination of PLL and GO, the PLL/GO/GCE shows the best electrocatalytic activity to the redox reactions of DA and UA (Figure 3E). It is found that the oxidation peak current of UA or DA at PLL/GO/GCE is much larger than the sum of relative those at GO/GCE and PLL/GCE, which means that the strong synergistic effect exists between PLL and GO.

For the PLL/GCE (Curve D), two redox peaks of DA appear at 280 and 257 mV, with a peak potential separation of 23 mV. These results suggest that PLL can benefit to the increase in electron transfer between DA and the modified electrode. The oxidation potentials of DA and UA were located at 0.280 and 0.408 V, respectively. While for the PLL/GO/GCE, the relative potentials of DA and UA shifted to 0.284 and 0.442 V, respectively. The increased overpotential of PLL/GO/GCE makes the difference between these two oxidation peak potentials increase from 0.128 V at PLL/GCE to 0.152 V at GO/GCE and to 0.158 V at PLL/GO/GCE, which facilitate the simultaneous detection of DA and UA. Thus, the result, i.e., the increase of peak current densities and difference of oxidation potentials, which may be attributed to the good combination of PLL with the more active adsorption sites and GO with a higher specific area and the possible synergistic effect of both composites, favors the simultaneous determination of DA and UA.

### 2.3. Effective Surface Area of the PLL/GO/GCE

The chronocoulometry technique was applied to estimate the electrochemical active surface areas of the bare GCE, PLL/GCE, untreated-GO/GCE and PLL/GO/GCE. According to the Anson equation [36] below,

*Q*(*t*) = 2*nFAcD*^1/2^*t*^1/2^π^−1/2^ + *Q_dl_* + *Q_ads_*

where *A* is the electrochemical active area of the working electrode, *F* is the Faraday constant, *c* is the concentration of the substrate, *D* is the standard diffusion coefficient in solution, *n* is the number of transfer electrons, *Q_dl_* is the double-layer charge, and other symbols have their acquiescent meanings.

**Figure 4:**
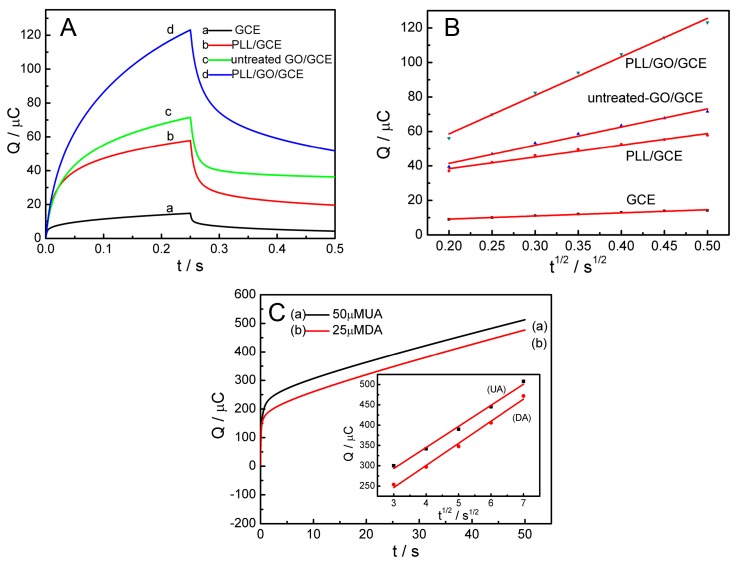
(**A**) Chronocoulometry curves for the bare GCE (a); PLL/GCE (b); untreated-GO/GCE (c) and PLL/GO/GCE (d) in 5.0 × 10^−4^ mol·L^−1^ [Fe(CN)_6_]^3−^ solution containing 0.1 mol·L^−1^ KCl. Initial potential, −0.2 V; final potential, 0.6 V; number of steps, 2; pulse width, 0.25 s; sample interval, 0.002; (**B**) linear relationship between *Q* and *t*^1/2^ for the PLL/GO/GCE, PLL/GCE, untreated-GO/GCE and the bare GCE; (**C**) Chronocoulometric curve of PLL/GO/GCE (a) for 50 μM UA and (b) for 25 μM DA in CPS (pH 5.0). Initial potential, −0.2 V; final potential, 0.6 V; number of steps, 2; pulse width, 50 s; sample interval, 0.002. The inset shows the linear relationship between the charge (Q) and square root of time (*t*^1/2^).

The relationship between charge (*Q*) and time (*t*) is shown in Figure 4A. The plot of *Q* versus *t*^1/2^ shows a linear relationship after background subtraction (Figure 4B). The effective electrochemical active area, *A*, can be estimated from the slope of *Q* versus *t*^1/2^. The slopes based on five parallel test results are 18.09, 68.14, 105.69, and 223.06 μC·s^−1/2^ for the bare GCE, PLL/GCE, untreated-GO/GCE and PLL/GO/GCE, respectively. When *n* = 1, *c* = 0.5 mM, and *D* = 7.6 × 10^−6^ cm^2^·s^−1^ (25 °C) [37], the effective surface areas of the bare GCE, PLL/GCE, untreated-GO/GCE and PLL/GO/GCE are calculated as 0.12, 0.45, 0.699 and 1.485 cm^2^, respectively. The effective electrochemical active area of the PLL/GO/GCE is twelve times, three times, and two times greater than that of the bare GCE, PLL/GCE and untreated-GO/GCE, respectively. The high active area of PLL/GO/GCE can increase the adsorption capacities of the modified electrodes toward UA and DA, leading to the enhancement of the current response at the PLL/GO/GCE. The large background current of the CV may also be attributed to the high electron transfer efficiency of the PLL/GO/GCE. In addition, the high surface area of the PLL/GO/GCE can also provide a strong evidence that the PLL and GO components were successfully immobilized on the GCE surface.

The chronocoulometry technique was also used to estimate the diffusion coefficient of UA and DA on the PLL/GO/GCE (Figure 4Ca,b). Where A is 1.485 cm^2^, c is the concentration of UA (5 × 10^−5^ M) or DA (2.5 × 10^−5^ M), the number of transfer electrons involved in the oxidation of UA or DA are 2 respectively. The calculated diffusion coefficients are 1.03 × 10^−5^ cm^2^/s and 4.54 × 10^−5^ cm^2^/s for UA and DA, respectively.

### 2.4. Influence of Scan Rate

**Figure 5:**
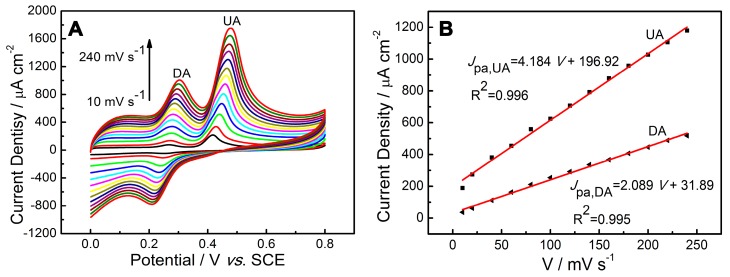
(**A**) CVs of 10.0 μΜ DA and 150.0 μΜ UA in CPS (pH 5.0) on the PLL/GO modified GCE with different scan rates ranging from 10 to 240 mV·s^−1^; (**B**) the dependence of *J*_pa_ of UA and DA on scan rate with different scan rates ranging from 10 to 240 mV·s^−1^.

We investigated the influence of scan rate on the peak currents of UA and DA. Figure 5A shows the CVs of 10.0 μM DA and 150.0 μM UA in CPS (pH 5.0) acquired at the PLL/GO-modified GCE with different scan rate ranging from 10 to 240 mV·s^−1^, the anodic peak current densities of UA and DA all increase linearly with the increase of scan rate (Figure 5B). This result indicates that the oxidation reactions of DA and UA at the PLL/GO/GCE electrode are an adsorption-controlled process. The linear regression equation relating current density *J*_pa_ to the scan rate (*v*) was found to be *J*_pa,UA_ (μA·cm^−2^) = 4.1841 *v*(mV·s^−1^) + 196.92 (R^2^ = 0.996) for UA and *J*_pa,DA_ (μA·cm^−2^) = 2.089 *v*(mV·s^−1^) + 31.89 (R^2^ = 0.995) for DA. Moreover, the redox peak potentials of DA shifted positively and negatively, respectively, as the scan rate increased, suggesting that the redox reaction of DA is quasi-reversible [38]. The anodic peak potential of UA shifted positively with increasing the scan rate. In addition, for the adsorption-controlled kinetic process, the interaction between the PLL or GO and the analyte molecules seems to be of importance. This comes from the intermolecular effects such as hydrogen bonds between PLL/GO and DA or UA.

### 2.5. Influence of pH

**Figure 6:**
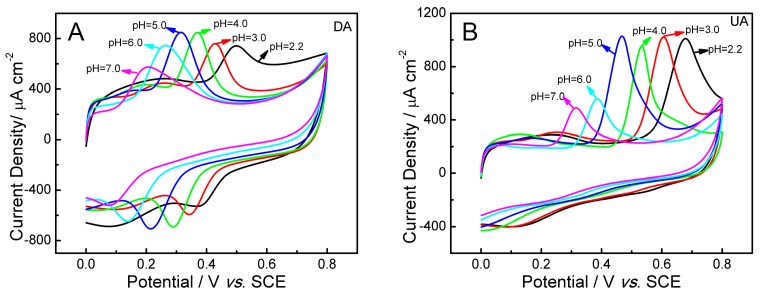

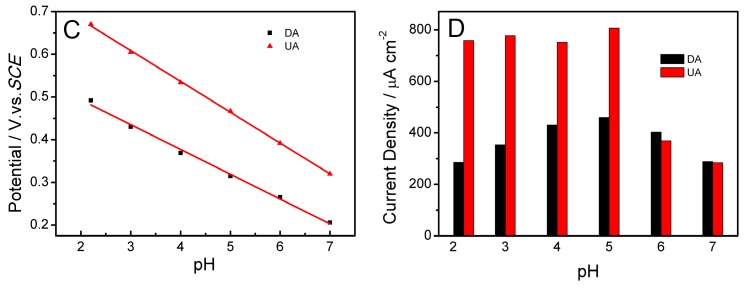
CVs at PLL/GO/GCE in CPS with different pH containing 25.0 μΜ DA (**A**) and 200.0 μΜ UA (**B**); Effect of pH value on the anodic peak potentials (**C**) and anodic peak current densities (**D**) of DA and UA.

**Scheme 1:**
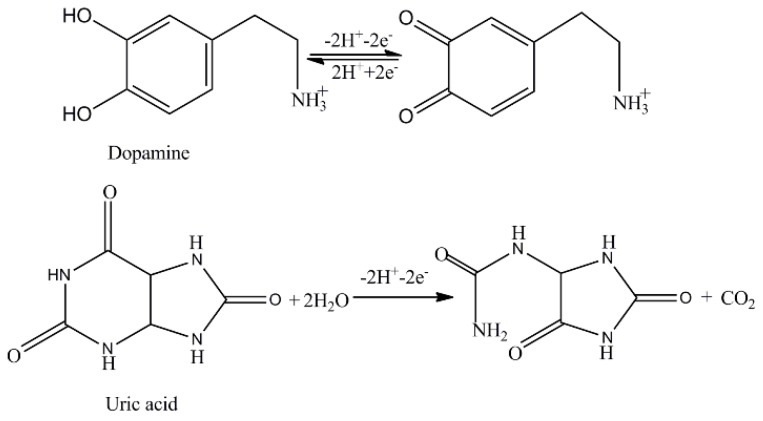
Probable oxidation mechanism for DA and UA.

The influence of buffer pH on the anodic peak potential of UA and DA was investigated by separately determining the CVs of the two compounds in CPS at a pH range of 2.2 to 7.0. Figure 6 illustrates the relationship of the oxidation peak potential and oxidation peak current density for UA and DA. The anodic peak potentials for UA and DA showed the same trends and became more negative over the measured pH range of 2.2–7.0, indicating that the oxidation of the two species at the PLL/GO/GCE is a proton-involved reaction [39]. The relationship between the potential and pH was linear, and the related regression equations were found to be *E*_pa,DA_ (V) = 0.6095–0.058 pH (R^2^ = 0.995) for DA and *E*_pa,UA_ (V) = 0.834–0.07399 pH (R^2^ = 0.995) for UA. The slopes were 58 and 73.99 mV pH^−1^ for DA and UA, respectively, which are close to the theoretical value of 59 mV·pH^−1^. It indicates that the electrochemical oxidation of UA and DA at PLL/GO/GCE involves an equal number of protons and electrons. Therefore, the possible reaction mechanisms of UA and DA at the proposed electrode are shown in Scheme 1 [40].

As can be seen in Figure 6D, the peak currents of UA and DA increased with increasing pH up to 5.0, then began to decrease upon further increasing the pH. The maximum value was observed at pH 5.0 for both UA and DA. To obtain better sensing performance, pH 5.0 was chosen as the optimal pH value for the following experiments.

### 2.6. Optimization of Preparation Conditions of PLL/GO/GCE Sensor

The amount of GO on GCE was optimized by varying its volume in the range of 2–6 μL (1 mg·mL^−1^), followed by electrochemical polymerization of PLL for 10 cycles. The response current density of DA was explored by differential pulse voltammetry (DPV) at various PLL/GO/GCEs. As shown in Figure S1A, the peak current density of DA increased with the volume of GO up to 5 μL, which is mainly attributed to the large surface area of the GO casted onto the GCE. Beyond that point, the peak current density of DA had no significant increase. As a result, 5 μL of GO (ca. 5 μg) was chosen to prepare the PLL/GO/GCE in this work.

The thickness of the PLL film was a crucial control factor for the oxidation of DA. The amount of PLL electrodeposited onto the surface of the untreated-GO/GCE was controlled by the CV scan. Figure S1B describes the relationship between the oxidation peak current density of DA at various PLL/GO/GCEs obtained at different scanning numbers of the electrochemical polymerization (GO, 5 μL 1 mg·mL^−1^). The peak signal successively increased with increasing electrodeposition scan number up to ten cycles, which is due to the improvement of the active sites in PLL/GO. Further increasing the polymerization cycles led to a decrease in the anodic peak current, suggesting that the PLL film was overly thick, consequently hindering the electron transfer rate and the mass transport process of DA. Therefore, the optimized number of electropolymerization scans was selected as ten for the formation of PLL films in the following experiments.

### 2.7. Detection of UA and DA

#### 2.7.1. Selective Detection

**Figure 7:**
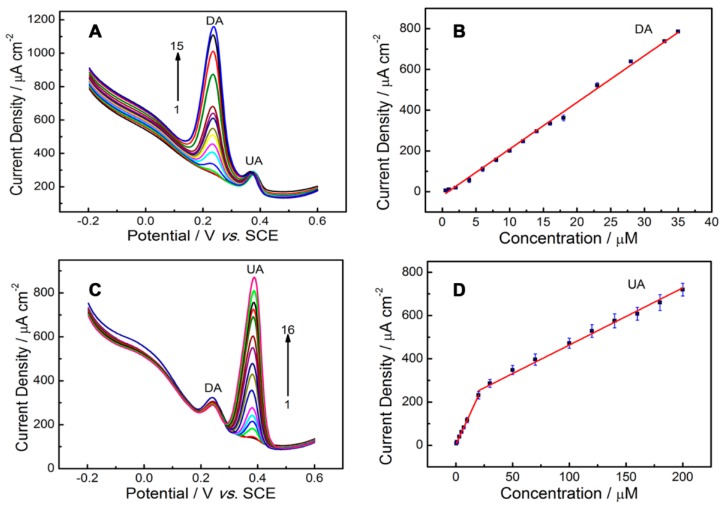
(**A**) DPV curves at PLL/GO/GCE in CPS of pH 5.0 containing 20 μM UA and different concentrations of DA (from 1 to 15): 0.5, 1, 2, 4, 6, 8, 10, 12, 14, 16, 18, 23, 28, 33, and 35 Μm; (**B**) The fitting curve of oxidation peak current density of DA versus the corresponding concentration of DA; (**C**) DPV curves at PLL/GO/GCE in CPS of pH 5.0 containing 5 μM DA and different concentrations of UA (from 1 to 16): 0.5, 1, 3, 5, 7, 10, 20, 30, 50, 70, 100, 120, 140, 160, 180 and 200 μM; (**D**) The fitting curve of oxidation peak current density of UA versus the corresponding concentration of UA.

The selective detection of DA and UA at the PLL/GO-modified GCE was investigated by DPV in a CPS solution containing a mixture of DA and UA. When the concentration of DA changed, the concentration of UA remained constant, and vice versa. As shown in Figure 7, the oxidation peak current density of DA responded linearly to its concentrations in the range of 0.5–35 μM in the presence of 5 μM UA. The calibration equation is *J*_pa,D_ (μA·cm^−2^) = 22.89 *C*_DA_ (μM) − 19.52 (R^2^ = 0.998). The oxidation peak current densities of UA remained nearly constant as the concentration of DA increased, suggesting that the addition of DA does not interfere with the detection of UA. Similar results were obtained during UA determination in the presence of 5 μM DA. The peak current density of DPV was proportional to the concentration of UA over the range of 0.5–20 μM and 20–200 μM, resulting in linear equations of *J*_pa,UA_ (μA·cm^−2^) = 11.41 *C*_UA_ (μM) + 3.14 (R^2^ = 0.998) and *J*_pa,UA_ (μA·cm^−2^) = 2.639 *C*_UA_ (μM) + 199.74 (R^2^ = 0.997), respectively. The calculated detection limits (LOD, 3S_b_/q, where S_b_ is the standard deviation of the blank signals, while q is the slope of the calibration curve.) were 0.018 and 0.031 μM for DA (0.5–35 μM) and UA (0.5–20 μM), respectively.

#### 2.7.2. Simultaneous Detection

**Figure 8:**
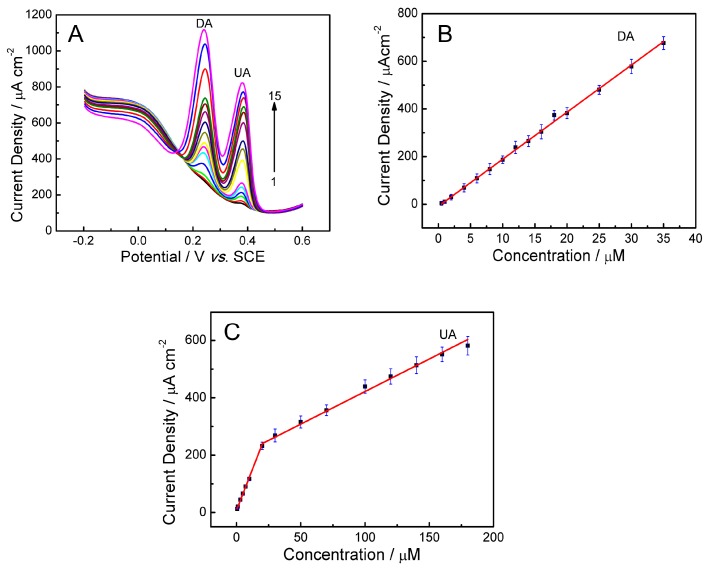
(**A**) DPV curves of DA and UA at PLL/GO/GCE in CPS of pH 5.0. Concentrations of the two compounds (from **1** to **15**): DA (0.5, 1, 2, 4, 6, 8, 10, 12, 14, 16, 18, 20, 25, 30, and 35 μM); UA (0.5, 1, 3, 5, 7, 10, 20, 30, 50, 70, 100, 120, 140, 160, and 180 μM); (**B**) The fitting curve of oxidation peak current density of DA versus the corresponding concentration of DA; (**C**) The fitting curve of oxidation peak current density of UA versus the corresponding concentration of UA.

DA and UA were simultaneously detected by changing their concentrations using the PLL/GO/GCE at pH = 5.0. When the concentrations of DA and UA changed synchronously, the oxidation peak current densities increase accordingly (Figure 8A). The results show that the simultaneous detection of DA and UA with two obviously peaks of 240 and 386 mV, corresponding to the oxidation of DA and UA, respectively, becomes possible at the PLL/GO/GCE. It can be seen from Figure 8B, the oxidation peak current density of DA is proportional to its concentration from 0.5 to 35 μM. The regression equation is *J*_pa,DA_ (μA·cm^−2^) = 19.72 *C*_DA_ (μM) − 0.61 (R^2^ = 0.998). Figure 8C shows two linear segments with different slopes for UA concentration; namely, for 0.50–20 μM the regression equation is *J*_pa,UA_ (μA·cm^−2^) = 11.50 *C*_UA_ (μM) + 7.93 (R^2^ = 0.996), while for 20.0–180 μM, the regression equation is *J*_pa,UA_ (μA·cm^−2^) = 2.27 *C*_UA_ (μM) + 194.72 (R^2^ = 0.992). The detection limits for DA and UA are 0.021 and 0.074 μM with the linear range or 0.5–35 and 0.50–20 μM, respectively. The sensitivities of PLL/GO/GCE for DA and UA in the mixture were 19.72 and 11.5 μA·cm^−2^·μM^−1^, while the sensitivity for individual DA or UA was 22.89 or 11.41 μA·cm^−2^·μM^−1^, respectively, indicating that the interference between DA and UA in the mixture could be ignorable. The PLL/GO/GCE can be utilized to simultaneously detect the DA and UA with good selectivity and high sensitivity.

The range of linearity, detection limit and sensitivity of this proposed method were compared with some other reports for DA and UA determination; the results were listed in Table 1. This clearly shows that the analytical parameters, such as sensitivity and detection limit of our work, are superior or comparable to the earlier reports.

### 2.8. Reproducibility, Stability and Interferences

**Figure 9:**
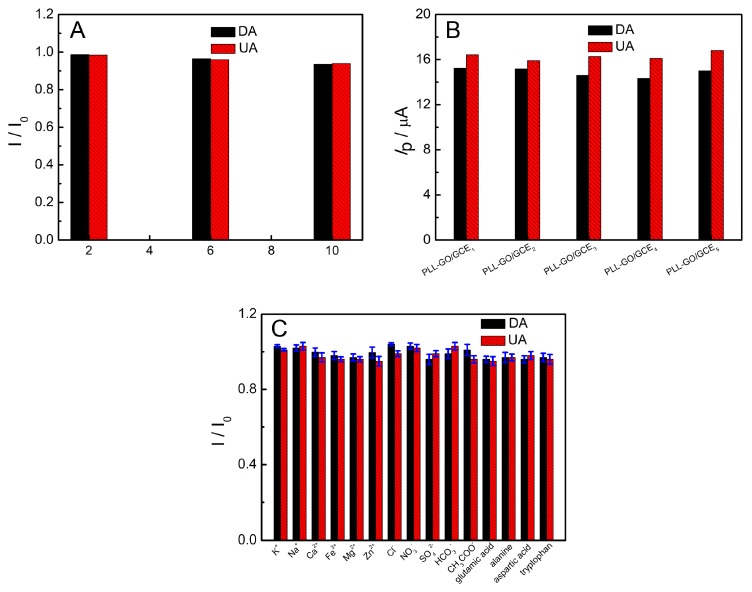
(**A**) Response of PLL/GO/GCE to the mixture of 10 μM DA and 100 μM UA at the 2nd, 6th and 10th day to compare the response at the first day; (**B**) The responses of five different modified electrodes with PLL and GO toward a mixture of 10 μM DA and 20 μM UA; (**C**) Interference study of inorganic ions and amino acids on response of 10 μM DA and 100 μM UA in pH 5.0 CPS buffer solution at PLL/GO modified GCE.

The stability and reproducibility of the PLL/GO/GCE were also studied. The stability of the constructed electrode was investigated by storing at 4 °C for 10 days. The DPV curves were measured and compared with the original curves. The results showed that the oxidation peak currents of DA and UA decreased less than 6.5%, indicating that the proposed electrode is very stable (Figure 9A). The repeatability of the PLL/GO/GCE was estimated by measuring the responses to five groups of independent solutions of the mixture of 10 μM DA and 20 μM UA. Each solution was measured for six times. The relative standard deviations were determined to be 4.5% and 5.3% for DA and UA, respectively. The reproducibility of the PLL/GO/GCE was also explored. Five different modified electrodes with PLL and GO and their responses toward a mixture of 10 μM DA and 20 μM UA were recorded (Figure 9B). The relative standard deviations were determined to be 2.6% and 2.1% for DA and UA, respectively. The results indicate that the modified electrode exhibits an acceptable repeatability and reproducibility.

The possible interferences of some inorganic ions on the determination of 10 μM DA and 100 μM UA were studied by DPV. The results showed that Na^+^ (100 mM), K^+^ (50 mM), Ca^2+^ (5 mM), Fe^3+^ (5 mM), Cl^−^ (100 mM), Zn^2+^ (5 mM), Mg^2+^ (5 mM), NO_3_^−^ (50 mM), SO_4_^2−^ (50 mM), HCO_3_^−^ (50 mM), CH_3_COO^−^ (50 mM), glutamic acid (400 μM), alanine (400 μM), aspartic acid (400 μM) and tryptophan (200 μM) did not interfere with the determination (signal changes less than 5%) (Figure 9C), indicating that the proposed electrode is highly selective.

**Figure 10:**
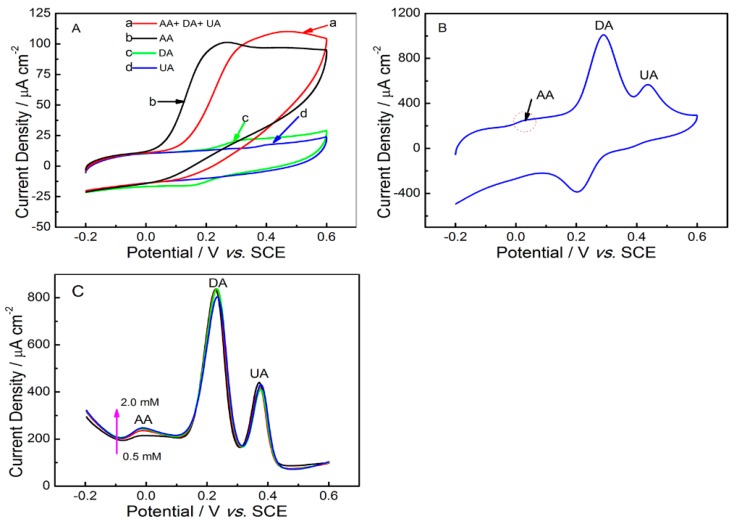
(**A**) CVs of different electroactive substances in CPS (pH 5.0) at bare GCE. (a) 500 μM ascorbic acid (AA) + 30 μM DA + 70 μM UA; (b) 500 μM AA; (c) 30 μM DA; (d) 70 μM UA. Scan rate: 100 mV·s^−1^; (**B**) Cyclic voltammogram of 500 μM AA + 30 μM DA + 70 μM UA in CPS (pH 5.0) at PLL/GO/GCE, Scan rate: 100 mV·s^−1^; (**C**) Differential pulse voltammetry (DPV) of AA with different concentrations (0.5 mM, 1.0 mM, 1.5 mM, 2.0 mM) in the present of 30 μM DA + 70 μM UA in CPS (pH 5.0) at PLL/GO/GCE.

AA always coexists with DA and UA in human body fluids, and the concentration of AA is much higher than that of UA and DA. The oxidation peak potential of AA is similar to that of DA and UA at the bare electrode. As shown in Figure 10A, the oxidation potentials of AA, DA and UA were located at 0.268, 0.32 and 0.40 V (vs. SCE), respectively. AA exhibits nearly an identical oxidation peak to that of DA (Figure 10Ab,c). Therefore, the presence of AA may interfere with the detection of DA and UA (Figure 10Aa). However, the PLL/GO/GCE can separate the peak potentials of AA, DA and UA (Figure 10B). Even when the concentration of AA was increased to the 2.0 mM, there was no obvious interference in the determination of DA and UA (signal change less than 5%, Figure 10C). Therefore, the proposed PLL/GO modified GCE can be used to determine UA and DA in the presence of high concentrations of AA, which is in accordance with the literature [41].

All of these advantages of the proposed sensor based on PLL/GO/GCE result from the combination of graphene and PLL, which takes full advantages of each of their merits and integrates their unique properties.

### 2.9. Analysis of Practical Samples

#### 2.9.1. Determination of UA in Real Biological Samples

DA and UA often coexist in real biological samples, such as serum and urine. Therefore, human blood serum and urine were chosen as the actual samples to investigate the possibility of the application of the proposed method by measuring the concentration of UA. All of the urine and blood serum samples were diluted by 100-fold and 5-fold, respectively, using CPS (pH 5.0). The standard addition technique was utilized to measure the recovery by the proposed DPV method. The summarized results for the analysis of human blood serum and urine samples are given in Table 2. The results show that the recovery of the proposed method is accurate and precise, indicating that the PLL/GO-modified GCE may be efficiently used to detect UA in blood serum and urine samples.

#### 2.9.2. Determination of DA in Dopamine Hydrochloride Injection

To ascertain the performance of the proposed approach for the determination of DA in dopamine hydrochloride injection, the PLL/GO/GCE was applied to the detection of DA in a pharmaceutical product. The sample was diluted by 5-fold with CPS (pH 5.0), and then a different dilution of the sample was added into the CPS (pH 5.0) for detection. The results are shown in Table 3. The estimated recovery value ranged from 99.5% to 102.6%. These satisfactory results indicate that the proposed method based on the PLL/GO/GCE is reliable for the detection of DA in dopamine hydrochloride injection.

## 3. Experimental Section

### 3.1. Reagents

Uric acid, dopamine and ascorbic acid were obtained from Aladdin Industrial Corporation (Shanghai, China). Graphite powder was purchased from Sinopharm Chemical Reagent Co., Ltd., Shanghai, China. All other chemicals were of analytical reagent grade. The CPS with different pH values was mixed with stock solutions of 0.2 M Na_2_HPO_4_ and 0.1 M citric acid. The PBS was prepared with the stock solutions of 0.2 M NaH_2_PO_4_ and 0.2 M Na_2_HPO_4_. De-ionized water with a resistivity of 18 MΩ cm was used throughout the experiments.

### 3.2. Apparatus

All electrochemical experiments were performed with a CHI660D electrochemical system (CH Instruments, Shanghai, China). The electrochemical measurements were performed in a single-compartment cell with a conventional three-electrode system, where a bare or modified GCE (CHI104, *d* = 3 mm), a platinum wire, and a saturated calomel electrode (SCE) are used as the working electrode, the counter electrode and the reference electrode, respectively. All potential values were reported with respect to the reference electrode. All measurements were performed in an electrolytic cell with 10 mL of solution. The solution was bubbled with high-purity nitrogen for 15 min to remove the oxygen, and a nitrogen atmosphere was maintained over the surface of the solution throughout the measurements. The pH measurements were made on a pHS-2C digital pH meter (Shanghai Dapu Instruments Co., Ltd., Shanghai, China). The EDS maps were recorded using an FEI Quanta 250F. All of the experiments were performed at ambient temperature (25 ± 0.5 °C).

### 3.3. Preparation of the Modified Electrodes

GO was synthesized from natural graphite powder using a modified Hummers method as described elsewhere [42]. Then, the obtained graphite oxide was ultrasonicated for 2 h and a homogeneous GO suspension was prepared. Prior to modification, the bare GCE was carefully polished with 0.3 and 0.05 μm aluminum oxide powder and then ultrasonicated in alcohol and de-ionized water for several minutes sequentially. The PLL/GO modified electrode was prepared by a two-step strategy. Firstly, a homogeneous suspension of GO with a concentration of 1.0 mg·mL^−1^ (5 μL) was dropped onto the surface of the cleaned GCE and dried at room temperature for two hours. The obtained electrode is denoted untreated-GO/GCE. Secondly, the PLL film was electropolymerized onto the surface of untreated-GO/GCE by cyclic potential sweeping from −0.8 V to 2.0 V in 0.1 M PBS (pH 8.0) containing 0.01 M l-lysine at a scan rate of 50 mV·s^−1^. The amount of PLL deposited was controlled by the cycling number. The resulting electrode is denoted PLL/GO/GCE. After polymerization, the modified electrode was rinsed with de-ionized water to remove any physically adsorbed substances and dried in air for the following experiments. For comparison, a PLL/GCE was prepared with a bare GCE by the same method. In addition, an untreated-GO/GCE was scanned for 10 cycles in 0.1 M PBS by cyclic potential sweeping from −0.8 V to 2.0 V at 50 mV·s^−1^. The electrode was named as GO/GCE, where GO was overoxidized during CV scanning.

### 3.4. Experimental Methods

CV and DPV measurements were performed with three electrodes in CPS. CV measurements were conducted under the following conditions: initial potential, −0.2 V; high potential, 0.6 V; low potential, −0.2 V; and scan rate, 100 mV·s^−1^. DPV measurements were recorded the following conditions: initial potential, −0.2 V; final potential, 0.6 V, pulse width, 0.05 s; pulse amplitude, 50 mV.

### 3.5. Sample Preparation

Blood samples of healthy volunteers were obtained from the Nanjing University of Science and Technology Hospital. Fresh human blood samples were centrifuged to remove precipitating materials for 20 min at 5000 rpm. The serum collected was diluted 5-fold with CPS (pH 5.0) and 10 mL of the diluent was placed in the electrolytic cell to determine UA by the proposed DPV method.

The human urine samples were collected and diluted 100-fold with CPS (pH 5.0) before measurement. No other pretreatment was performed. The dopamine hydrochloride injection (Shanghai Harvest Pharmaceutical Co. Ltd., Shanghai, China) was purchased from the Nanjing University of Science and Technology Hospital. The sample was diluted 5-fold with CPS (pH 5.0).

## 4. Conclusions

We fabricated a PLL/GO-modified electrode by the electrochemical polymerization of l-lysine on the surface of GO sheets, which was successfully used as a sensory platform for the fast simultaneous detection of DA and UA. The calculated diffusion coefficients are 1.03 × 10^−5^ cm^2^/s and 4.54 × 10^−5^ cm^2^/s for UA and DA, respectively. The PLL/GO-modified GCE exhibited high electrocatalytic activity toward the electro-oxidation of DA and UA by significantly amplifying the peak currents. The larger peak separation between DA and UA achieved by CV or DPV at the PLL/GO/GCE allowed for their simultaneous detection. The proposed sensor showed acceptable sensitivity, good reproducibility, long-term stability, good selectivity and negligible ascorbic acid interference. Moreover, the modified electrode was also successfully utilized to the analysis of DA and UA in real samples with satisfactory results.

## Figures and Tables

**Table 1:** Determination of UA in human blood serum and urine samples (*n* = 3).

Electrode	Analyte	Linear Range (μM)	Detection Limit (μM)	Sensitivity (μA·cm^−2^·μM^−1^)	References
Nitrogen doped graphene/GCE	DA	0.5–170	0.28	0.45	[13]
	UA	0.1–20	0.045	20.61	
ERGO^a^/GCE	DA	0.5–60	0.5	7.11	[16]
	UA	0.5–60	0.5	7.97	
Poly(Vinyl alcohol)	DA	2–70	1.4	13.52	[24]
	UA	2–50	0.6	43.82	
Au/Reduced graphene oxide/GCE	DA	6.8–41	1.4	14.08	[17]
	UA	8.8–53	1.8	7.04	
PLL/GO/GCE	DA	0.5–35	0.021	19.72	This work
	UA	0.5–20 20–200	0.074	11.50	

ERGO^a^: electrochemically reduced graphene oxide.

**Table 2:** Determination of UA in human blood serum and urine samples (*n* = 3).

Sample	Found (μM)	Added (μM)	Found (μM)	Recovery (%)	R.S.D (%)
Serum 1	48.26	10	58.4	101.4	2.4
Serum 2	56.57	20	75.87	96.5	3.1
Urine 1	45.17	20	64.30	95.65	1.4
Urine 2	40.46	15	56.05	103.9	1.8

**Table 3:** Determination of dopamine hydrochloride injection (*n* = 5).

DA Specified (μM)	Added (μM)	Found (μM)	Recovery (%)	R.S.D (%)
10.5	0	10.25	-	1.86
10.5	5	15.63	102.6	1.07
10.5	10	20.45	99.5	0.83
10.5	15	25.67	101.1	1.21

## Supplementary Materials

The following are available online at www.mdpi.com/2079-4991/6/10/178/s1.
