# Supplementary material for: Simultaneous Detection of Dopamine and Uric Acid Using a Poly(l-lysine)/Graphene Oxide Modified Electrode
Source: Nanomaterials (Basel). 2016 Sep 28;6(10):178. doi: 10.3390/nano6100178 (PMC5245187; doi:10.3390/nano6100178)
Supplement: Supplementary file 1 [file nanomaterials-06-00178-s001.pdf]

Supplementary Materials: Simultaneous Detection of Dopamine and Uric Acid Using a Poly(L-lysine)/Graphene Oxide Modified Electrode

Yuehua Zhang, Wu Lei, Yujuan Xu, Xifeng Xia and Qingli Hao

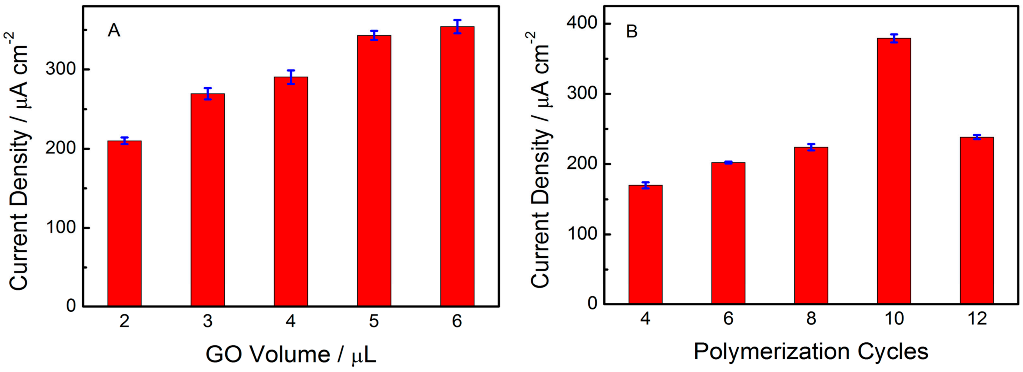

Figure S1. Effects of the content of GO suspension (**A**) and polymerization cycles of PLL (**B**) on the peak current of DA (20.0 μM) in pH 5.0 CPS (n = 5).
